# Auxin regulates functional gene groups in a fold-change-specific manner in *Arabidopsis thaliana* roots

**DOI:** 10.1038/s41598-017-02476-8

**Published:** 2017-05-30

**Authors:** N. A. Omelyanchuk, D. S. Wiebe, D. D. Novikova, V. G. Levitsky, N. Klimova, V. Gorelova, C. Weinholdt, G. V. Vasiliev, E. V. Zemlyanskaya, N. A. Kolchanov, A. V. Kochetov, I. Grosse, V. V. Mironova

**Affiliations:** 1 Institute of Cytology and Genetics SB RAS, Novosibirsk, Russia; 2 LCT&EB, Novosibirsk State University, Novosibirsk, Russia; 3 Ghent University, Department of Physiology, Laboratory of Functional Plant Biology, Ghent, Belgium; 4 Institute of Computer Science, Martin-Luther-University Halle-Wittenberg, Halle, Germany; 5 German Centre for Integrative Biodiversity Research (iDiv), Halle-Jena-Leipzig, Leipzig, Germany

## Abstract

Auxin plays a pivotal role in virtually every aspect of plant morphogenesis. It simultaneously orchestrates a diverse variety of processes such as cell wall biogenesis, transition through the cell cycle, or metabolism of a wide range of chemical substances. The coordination principles for such a complex orchestration are poorly understood at the systems level. Here, we perform an RNA-seq experiment to study the transcriptional response to auxin treatment  within gene groups of different biological processes, molecular functions, or cell components in a quantitative fold-change-specific manner. We find for *Arabidopsis thaliana* roots treated with auxin for 6 h that (i) there are functional groups within which genes respond to auxin with a surprisingly similar fold changes and that (ii) these fold changes vary from one group to another. These findings make it tempting to conjecture the existence of some transcriptional logic orchestrating the coordinated expression of genes within functional groups in a fold-change-specific manner. To obtain some initial insight about this coordinated expression, we performed a motif enrichment analysis and found cis-regulatory elements TBX1-3, SBX, REG, and TCP/site2 as the candidates conferring fold-change-specific responses to auxin in *Arabidopsis thaliana*.

## Introduction

Conventionally applied to genome-scale data, clustering procedures compare gene expression levels to detect gene clusters with similar expression patterns^1^. These gene expression clusters usually tend to be significantly enriched for specific functional categories. However, further implication is unclear: do the genes participating in the same biological process change their expression levels concordantly? If so, do the amplitudes of response to new conditions differ for various processes?

To answer these questions, we chose the model plant *Arabidopsis* as it has one of the best-annotated genomes among multicellular organisms. Auxin (indole-3-acetic acid, IAA) treatment was chosen as a stimulus since auxin response is one of the best-studied pathways in plants^2, 3^. Since auxin has a major role in root development^4, 5^ and auxin-induced transcriptome changes peak at approximately 6 h after treatment^6, 7^, we performed RNA-Seq on the roots of 6 h IAA-treated seedlings. RNA-Seq has higher accuracy and can estimate larger amplitudes of gene expression values^8–10^ than microarrays.

The functional annotation procedure uses three Gene Ontology (GO) controlled vocabularies (Biological Process, Molecular Function or Cell Compartment) and assesses overrepresented GO terms in the gene lists. This procedure is embodied in bioinformatics resources such as DAVID^11^ and AgriGO^12^. Functional annotation is routinely applied to: (1) lists of differentially expressed genes in a dataset with fold changes above a threshold, or (2) gene clusters united by certain expression patterns over a number of datasets. Here, we suggest combining both approaches to functionally annotate the genes, which differ by the response amplitudes within a single dataset. This combined approach would allow identifying if there are GO terms specifically enriched for the genes responding to auxin coordinatively, within a certain interval of fold changes, comparing the whole list of differentially expressed genes. For this procedure, we implement a bioinformatics algorithm and apply it to generated auxin responsive root transcriptome. To validate the results, we apply the same method to publicly available microarray data on auxin-induced transcriptomes examined over time-course^7^. Finally, we determine if there are cis-regulatory elements specifically overrepresented in the groups of differentially expressed genes (DEGs) responding to auxin in different fold change intervals.

## Results

### RNA-Seq analyses of auxin-induced transcriptome in *A. thaliana* roots and qPCR validation

To study the late auxin response in roots on a transcriptional level, we treated 3-day-old *A. thaliana* seedlings with 1 µM IAA for 6 h. The root transcriptome changes were analyzed by RNA-Seq (see Materials and Methods). Mapping of the RNA-Seq reads resulted in the detection of 20423 transcripts, including 88 from plastid and 120 from mitochondrial genomes. Through differential expression analysis, we found 789 genes significantly upregulated (UG) and 659 genes downregulated (DG) by auxin (false discovery rate [FDR] adjusted *p* < 0.05) (Supplementary Table 1). Fold change values for UGs and DGs varied in the ranges of 1.5–88 and 1.4–143, respectively. We detected the changes in transcript levels in response to auxin uniquely for nuclear genes and not for plastid or mitochondrial ones.

**Figure 1:**
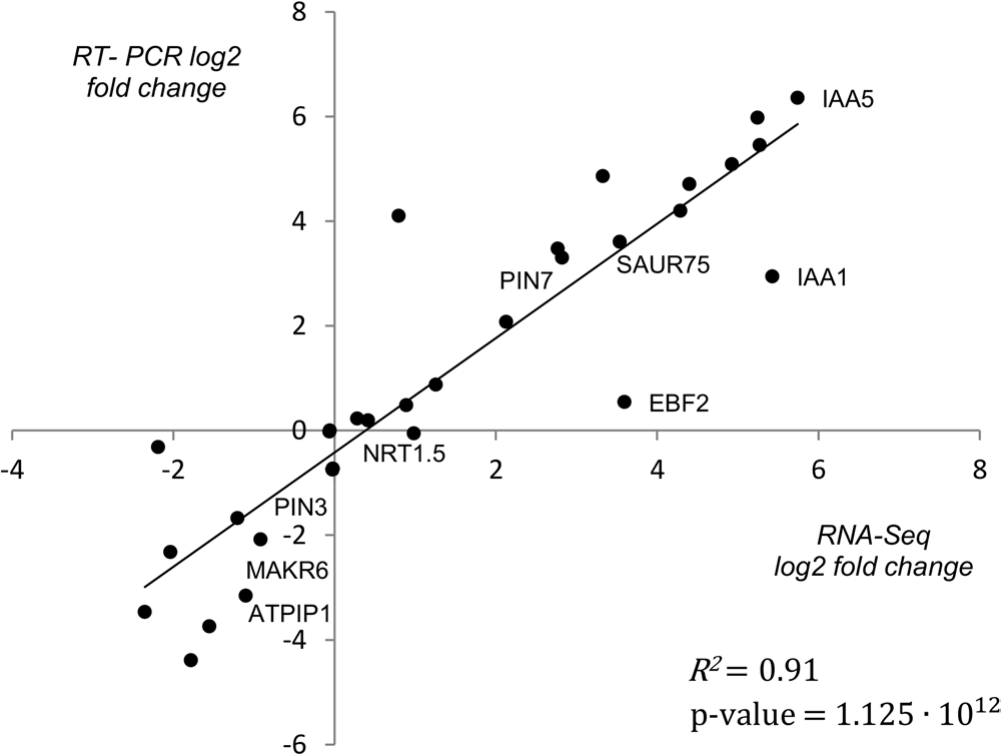
Scatterplot for log_2_ fold changes of 30 genes in RNA-Seq and qPCR experiments. Both log_2_ fold changes were well correlated (*R* ^*2*^ = 0.91, *p* < 5.0E-12, Supplementary Table 2).

We verified the RNA-Seq data by qPCR for 30 genes (Supplementary Tables 2 and 3). The Pearson correlation coefficient (R^2^ = 0.91, *p* < 5.0E-12) between the log_2_ fold changes in the gene expression levels shown by qPCR and RNA-Seq data (Fig. 1) suggested that the auxin response fold changes in the RNA-Seq experiment were sufficiently accurate.

### The fold-change-specific responses to auxin

To study how auxin coordinates transcription of the genes involved in the same biological processes, we needed to develop an accurate procedure (detailed in Supplementary Text).

**Figure 2:**
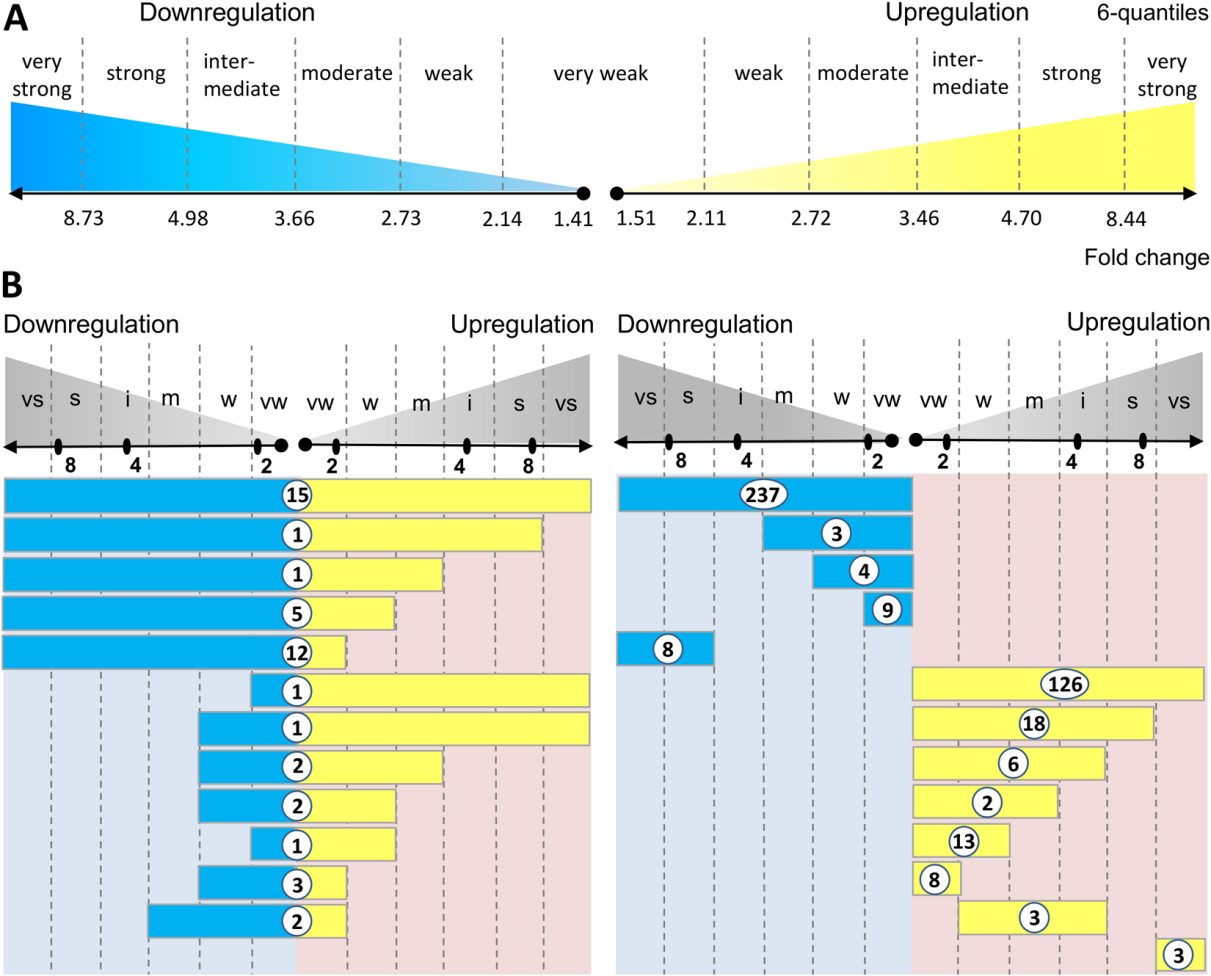
Assessment of fold-change-specific auxin response in auxin-reprogrammed transcriptome (6 h; 1 µM IAA). (**A**) Scale for the fold change intervals with the threshold values to subdivide UGs and DGs into 6 equal in size gene groups (6 quantiles). (**B**) The fold change intervals for UGs (yellow boxes) and DGs (blue boxes) associated with specific regulation of genes detected at least for one GO term. Each GO term was associated with only one fold change interval (either a single quantile or combination of neighboring quantiles) possessing the most significant enrichment (for UGs and DGs separately). The shortest intervals correspond to the tightest fold-change-specificity of auxin response. The longest intervals (from very weak to very strong) are not fold-change-specific. The numbers in the boxes indicate the amount of GO terms associated with auxin regulation within the fold change interval. On the left panel, blue and yellow boxes in one line correspond to the same set of GO terms. vs – very strong, s – strong, i – intermediate, m – moderate, w – weak, vw – very weak.

First, we ranked UG and DG lists separately by their fold changes and subdivided each list into six groups (6-quantiles) of equal size. We denominated groups as very weak, weak, moderate, intermediate, strong and very strong responses (Fig. 2A). Using the AgriGO SEA tool^12^, we functionally annotated the genes belonging to each quantile, various combinations of neighboring quantiles, and the overall DEG set (21 groups in total for UGs or DGs). For further analysis, we selected the GO terms with adjusted p-values less than 0.001 (Fisher’s exact test by SEA, Bonferroni corrected for the number of GO terms and the number of tested intervals).

We found 225 and 307 GO terms enriched in at least one tested fold change interval for UGs and DGs, respectively (Supplementary Table 4). Approximately 15% of these GO terms (36 in UGs and 45 in DGs) appeared significantly enriched in only the specific fold change intervals, indicating that auxin regulates some biological processes with a fine precision.

Moreover, we noted that for some GO terms, the *p-value* for a specific fold interval differed by several orders of magnitude from the *p-value* for the whole UG or DG sets. For example, the GO term “translation” was enriched for UGs with *p-value* of 7.3 × 10^−19^, but for very weak and weak responses, the *p-value* was many magnitudes lower (*p-value* = 6.6 × 10^−54^) (Table 1; Supplementary Table 4). To estimate the significance for such differences, we needed to apply a second step in our procedure, where we analyzed enrichment of the GO term by the genes activated (or inhibited) by auxin in a specific fold change interval versus the whole sets of UGs or DGs. This analysis was performed for each of 20 component fold change intervals. If the adjusted *p-value* in this comparison (Fisher’s exact test, Bonferroni correction for the number of selected GO terms and 20 intervals, see Materials and Methods) was less than 0.05, we considered the genes associated with the GO term as fold-change-specifically regulated by auxin in this selected interval (such GO term was defined as fold-change-specific).

**Table 1:** Contingency table for the estimation of fold-change-specificity of a GO term.

	The number of UGs in the fold change interval	The number of UGs outside the fold change interval	Total number of UGs	The portion of UGs in the fold change interval
The UGs belonging to the GO term	*96*	*12*	*108*	88.9%***
The rest UGs	*167*	*514*	*681*	*24.6%*
Total number of UGs	*263*	*526*	*789*	*33.3%*

An example of a “translation” GO term and a very weak to weak fold change interval.

We found that notable numbers of functional gene groups, 82 (36%) and 36 (12%), were fold-change-specific in UGs and DGs, respectively (Supplementary Table 4, Fig. 2B). The remaining GO terms (143 for UGs and 271 for DGs) were not specific in fold of response. In the following sections, we will describe the fold-change-specific groups in detail.

### Coherent auxin regulation of the genes whose products localize in the same cellular compartments or have similar molecular functions

It could be expected that most of the fold-change-specific GO terms should belong to the “molecular functions” GO vocabulary because annotation lists contain paralogs that often express redundantly. Instead, only a few GO terms from this vocabulary were associated with fold-change-specific response to auxin (Supplementary Fig. 1; Supplementary Table 4). Namely, the genes corresponding to GO term “Translation factor activity, nucleic acid binding” and the related terms “structural constituent of ribosome” and “structural molecule activity” were weakly upregulated. “Binding”, “RNA binding” and “nucleotide binding” terms were enriched among the genes with very weak to strong responses to auxin. Only the genes encoding enzymes with hydrolase activity were downregulated in a fold-change-specific window from very weak to moderate levels. Auxin affected expression of genes with many other molecular functions as well, but without fold-change-specificity (Supplementary Table 4).

**Figure 3:**
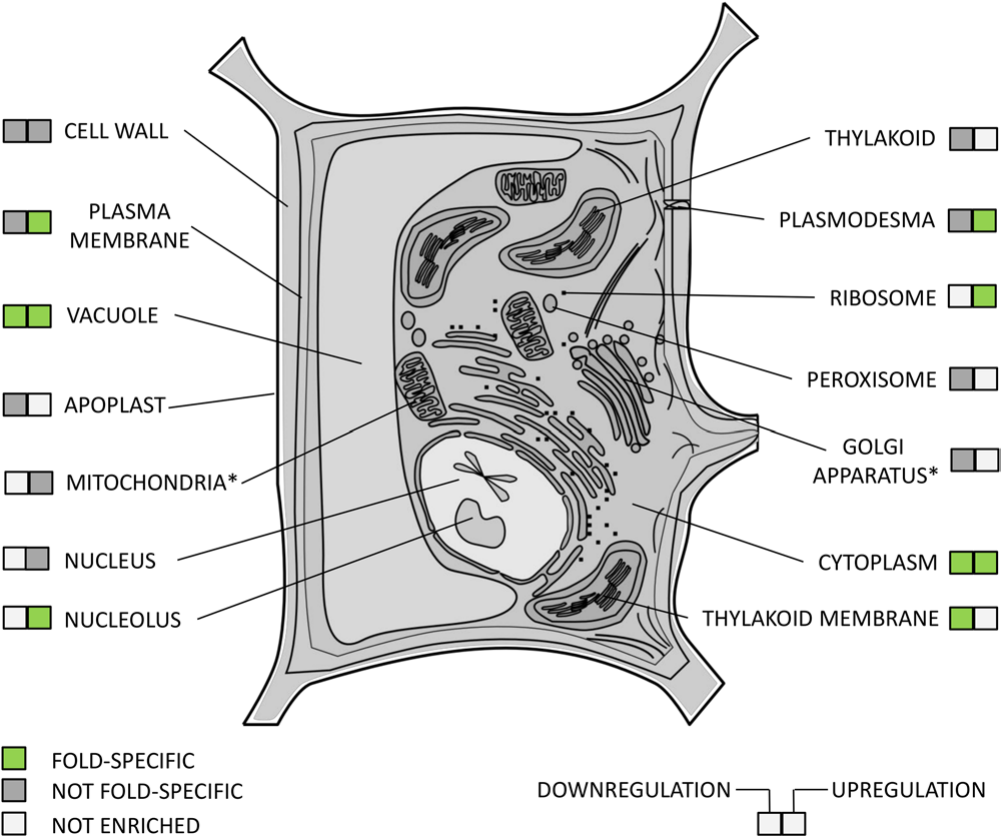
Cellular components differentially and fold-change-specifically regulated by auxin in the root. The source data are depicted in Supplementary Fig. 2 and Supplementary Table 4. *Golgi apparatus and mitochondria were included into the scheme since their biogenesis was auxin-regulated (Fig. 4).

In turn, when annotating auxin-responsive genes associated with cell components, a great heterogeneity of the related gene groups was expected, which might not be favorable to precise fold-change-specific regulation. Against this logic, cell component GO terms showed the most significant fold-change-specific response to auxin (Supplementary Table 4). Auxin specifically and differentially regulated the genes whose products localize to most cellular components (more than 70% of GO terms are fold-change-specific), except the Golgi apparatus, endoplasmic reticulum and mitochondria (Fig. 3, Supplementary Fig. 2). These results suggest that auxin coherently regulated the genes functioning in the same cellular compartment, changing their expression levels not only unidirectionally but also with the same amplitude of response. The genes encoding ribosomal RNA and ribosomal proteins were most tightly regulated by auxin.

### Summary of the processes fold-change-specifically activated/inhibited by auxin in the root

**Figure 4:**
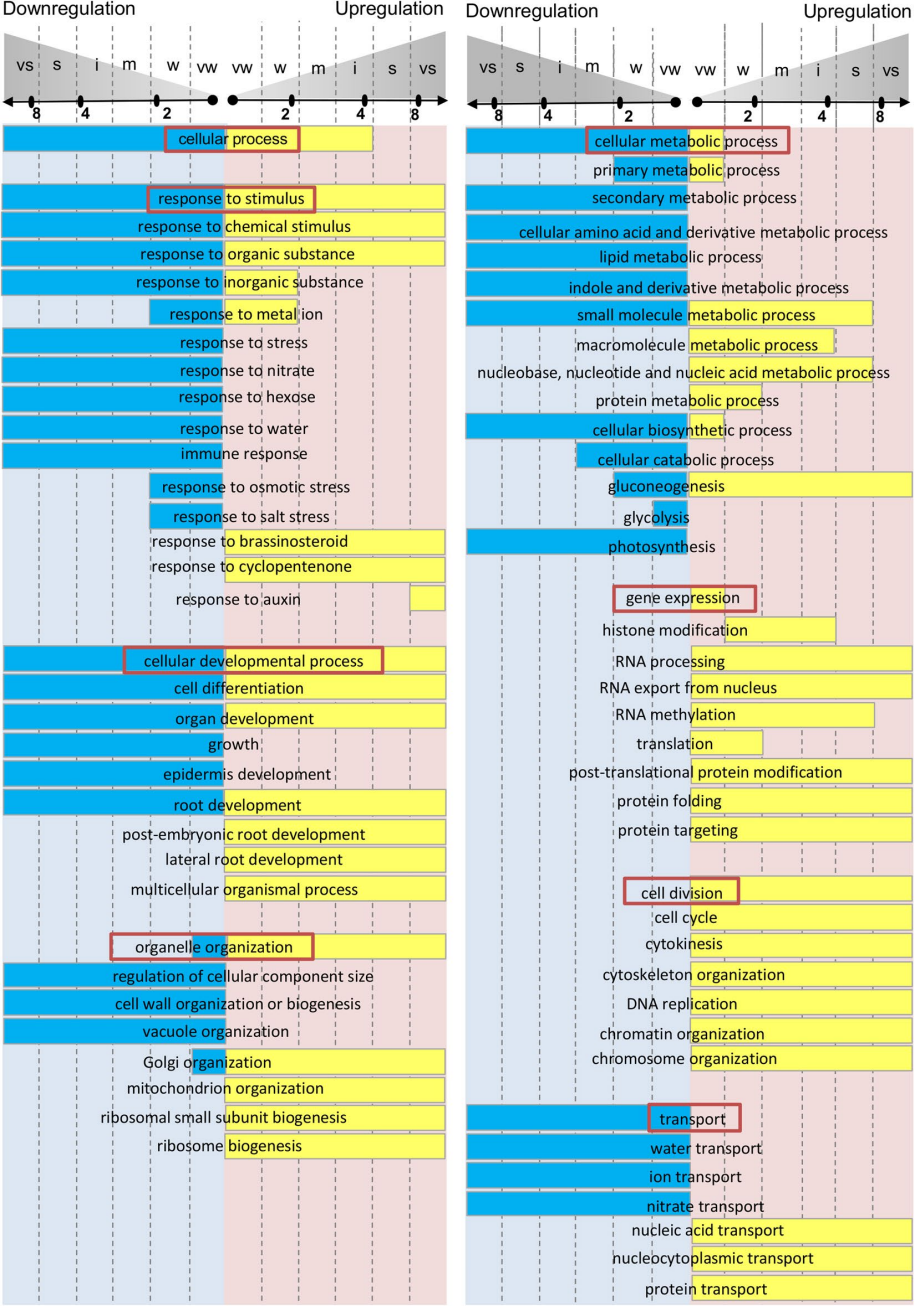
A general scheme of auxin-regulated biological processes in the root revealed in this study. For a particular GO term it shows the fold change interval in which significant part of belonging genes responded to auxin. The longest intervals (from very weak to very strong) are not fold-change-specific, while shorter ones are fold-change-specific. Activation of transcription shown by yellow boxes, inhibition of transcription by blue boxes. The representative GO terms were selected from the complete list of significantly enriched in auxin response GO Biological Processes (Supplementary Table 4). Red rectangular highlights the common term for the GO terms group. vs – very strong, s – strong, i – intermediate, m – moderate, w – weak, vw – very weak.

Although annotations of auxin-induced transcriptomes have been previously described^3, 6, 7^, applying a fold-change-specific approach makes the auxin regulation map more comprehensive. Based on the results of functional annotation of the RNA-Seq data in the knowledge domain of biological processes, we can propose a general scheme of auxin action in the root cell (Fig. 4). We subdivided auxin-responsive processes into seven basic groups: response to stimulus, cellular developmental process, cellular metabolic process, organelle organization, transport, cell division and gene expression. Auxin exclusively activated only two of these processes: cell division not fold-change-specifically and gene expression very weakly. Cell division, cell cycle, cytokinesis, DNA replication, cytoskeleton, chromatin, and chromosome organization – all these energy-consuming processes – were upregulated by auxin. For the remaining biological processes, auxin realizes a well-balanced and thrifty strategy that allows a cell to save its resources during division. Auxin moderately downregulated the catabolic processes and non-fold-specifically downregulated secondary metabolism, transport, and a number of other responses (Fig. 4). However, its influence on primary metabolism is more complex and fold-change-specific. In general, auxin inhibited all main metabolic processes except nucleic acid, protein and small molecule metabolism (the latter probably as an energy source for the former two processes). Auxin upregulated all three processes in a well-coordinated way so that significant numbers of the genes involved were activated with the same strength.

**Figure 5:**
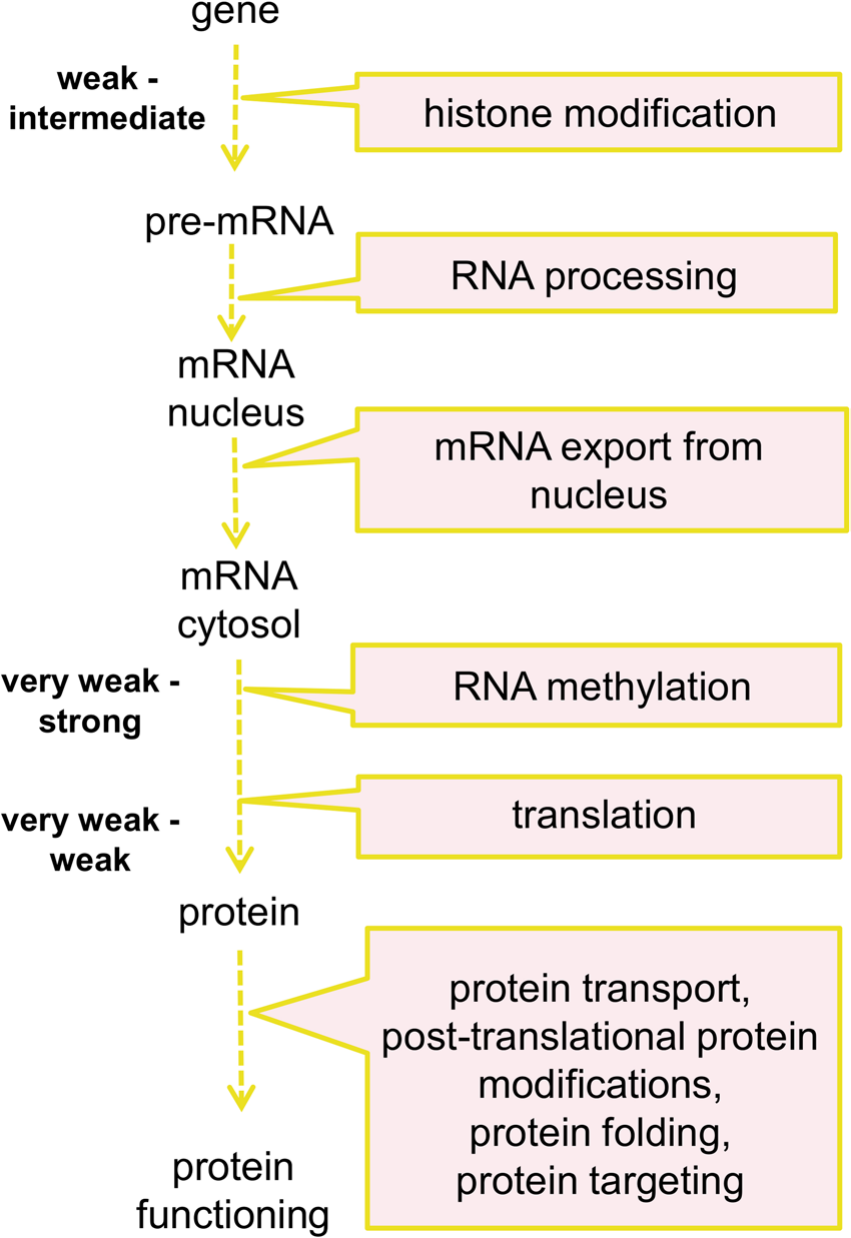
Gene expression control by auxin. All processes related to gene expression were upregulated in the root. Three bottlenecks are fold-change-specifically regulated by auxin, with the translation step regulated most tightly (very weak to weak levels). The source data are depicted in Fig. 4 and Supplementary Table 4.

This coordination was also true for gene expression processes as there were three stages where auxin activation occurred within the fold-change-specific window – histone modification (from weak to intermediate), translation (up to a weak level) and RNA methylation (up to a strong level). In accordance with the findings for cell components and molecular functions (Supplementary Figs 1 and 2), translation was the most tightly upregulated by auxin, indicating that protein synthesis can be a bottleneck in gene expression machinery (Fig. 5).

### Validation of fold-change-specific auxin response using independent data

To test if the fold-change-specific regulation of genes from the same pathway was not an artifact of our RNA-Seq data, we analyzed independent whole-genome data: time course microarray experiments on exogenous 1 µM IAA treatment in root^7^. The numbers of DEGs for either early (up to 2 h) or very late (24 h) auxin response were insufficient to proceed with our approach. Thus, we applied the two-step analysis to each of 4-, 8- and 12-h time point datasets separately (Supplementary Table 6).

**Figure 6:**
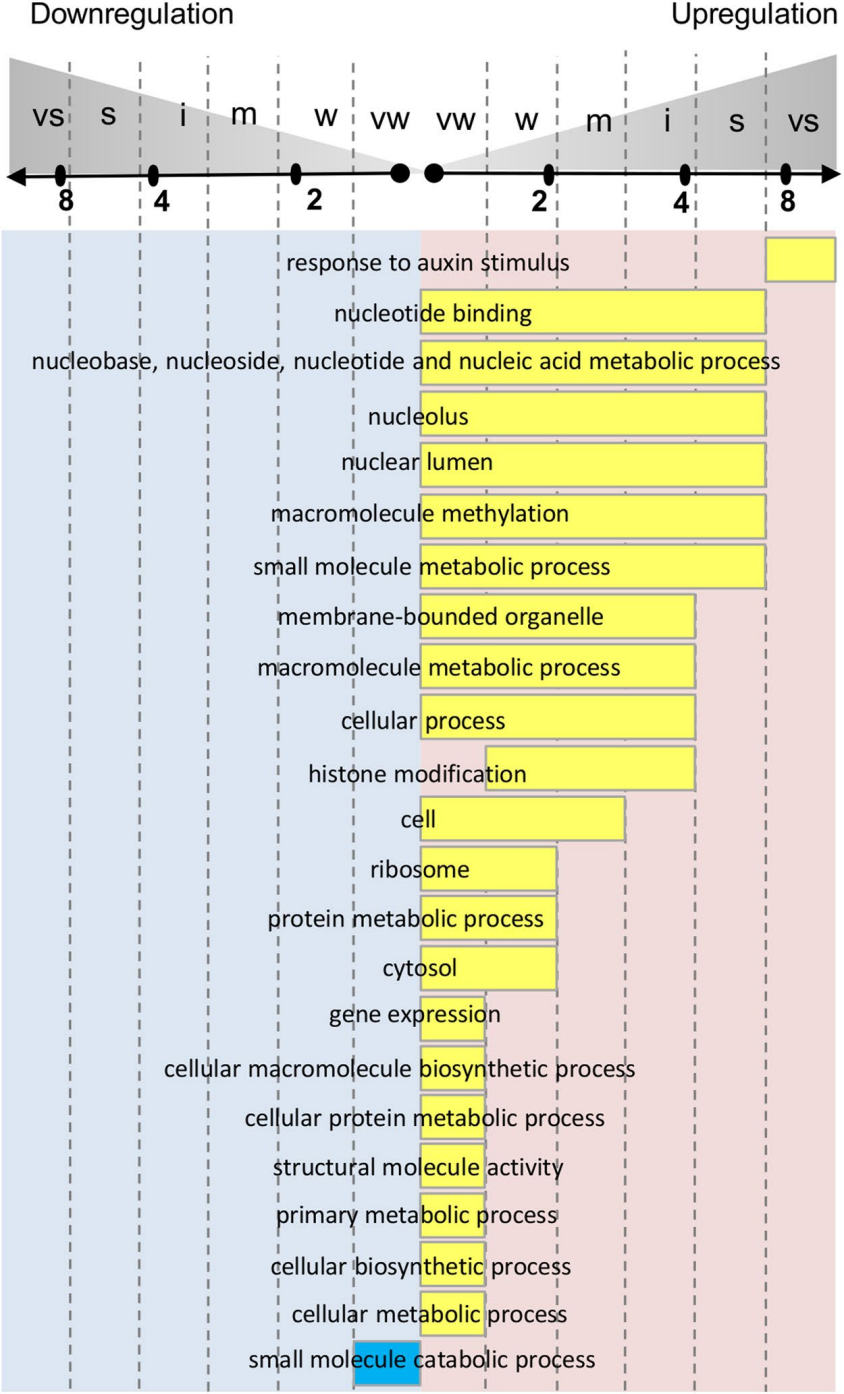
The functional gene groups fold-change-specifically responded to auxin in two independent experiments (current work and Lewis *et al*.^7^). Specific fold change intervals are depicted from RNA-Seq data and are designated in Figs 2 and 4. For details see Supplementary Tables 6 and 7.

In agreement with the RNA-Seq analysis, many processes fold-change-specifically responded to auxin in the microarray data^7^. Altogether, 364 GO terms were enriched among DEGs for 4-, 8- and 12-h time points (Supplementary Tables 6 and 7), and 63% of these terms matched those for our RNA-Seq experiment. Notably, 77% of fold-change-specific GO terms identified in the RNA-Seq experiment were also fold-change-specific in the microarray data (Supplementary Table 7). Figure 6 shows nonredundant list of independently confirmed fold-change-specific functional groups. The fold changes were in the same window or lower in the microarray data, which is meaningful because RNA-Seq allows the estimation of larger amplitudes of gene expression values compared to microarray analysis^8–10^.

This analysis independently confirms that functional categories stratify by the amplitude of fold changes through auxin transcriptional response. From them, only one category, ‘response to auxin stimulus’, with quick and very strong upregulation was most frequently reported as enriched in DEGs^3, 6, 7^, while the rest functional groups (Supplementary Table 4) has not been associated with specific amplitudes of response. These findings are particularly important because fold-change-specific responses with lower magnitude are much more significant and regulate a much greater number of genes (Supplementary Table 4).

### cis-regulatory elements responsible for fold-change-specific auxin responses

Next, we investigate to which degree the observed fold-change-specific auxin responses might be explained by association with potential cis-regulatory elements. The fast primary auxin response is mediated through the ARF family of transcriptional factors, which binds TGTC-containing cis-regulatory elements^13^. While several TGTCNN hexamers are associated with auxin-responsive gene expression in general^14^, we investigate here if there are motifs associated with fold-change-specific auxin response.

**Figure 7:**
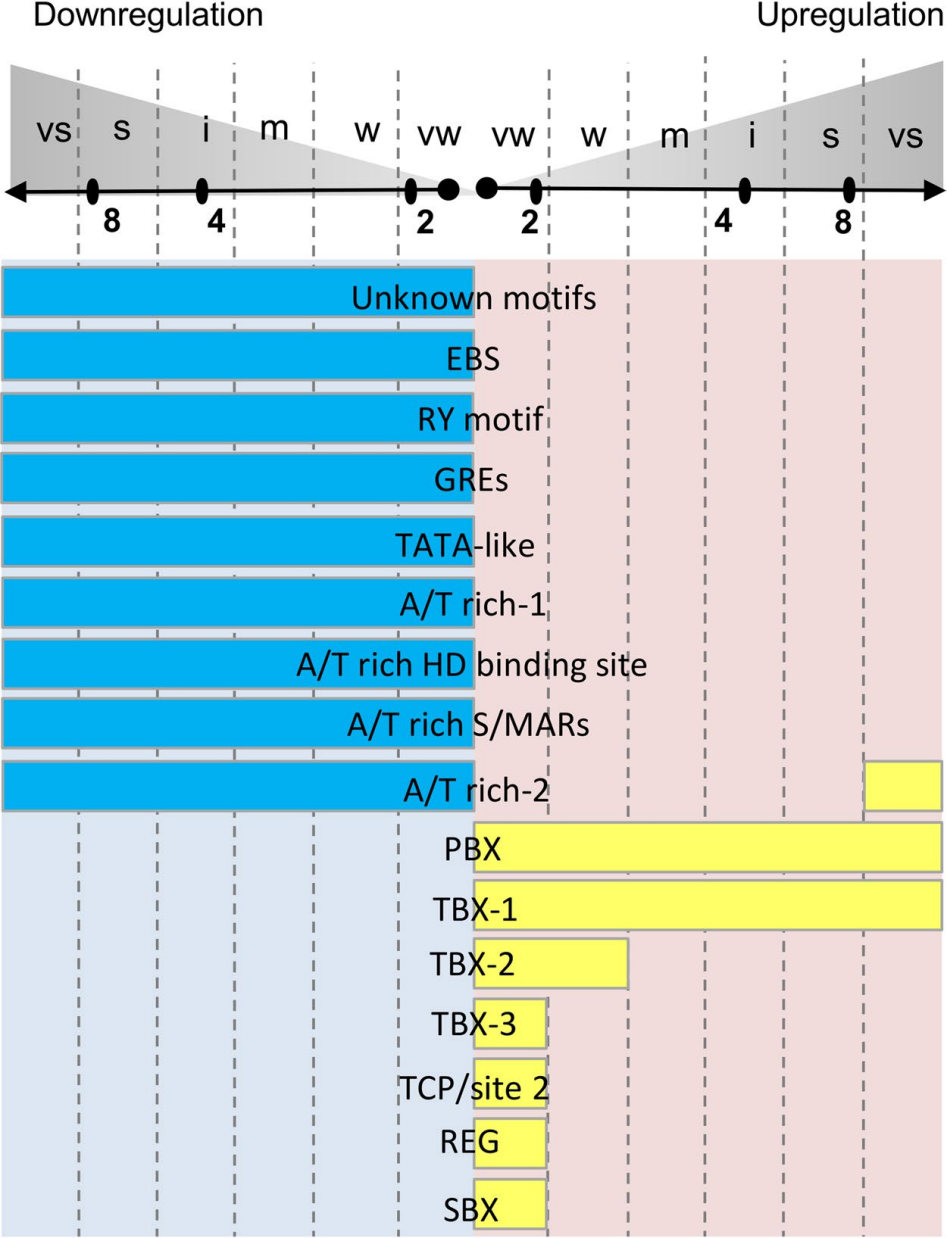
cis-Regulatory motifs found enriched in the upstream regions of the genes responded to auxin within certain fold change intervals. Unknown motifs (atacta/tagtat, atagta/tactat; atatga/tcatat, atatgt/acatat, taatag/ctatta, tatgta/tacata); EIN3-binding site EBS (atacat/atgtat)^30^; B3-family binding RY motif (tgcatg/catgca)^31^; bZIP-binding GRE/ABRE motifs (tacgtg/cacgta; acacgt/acgtgt, acgtgg/ccacgt)^32^, TATA-like (aatata/tatatt, actata/tatagt, atataa/ttatat, atatac/gtatat, atatag/ctatat, tataac/gttata, tatata, tatatg/catata; atatat)^33^; A/T rich-1 (aataat/attatt, aattat/ataatt, ataata/tattat, attaat, attata/tataat, taaata/tattta, tattaa/ttaata); A/T-rich Homeodomain (HD) binding site (taatta)^34^; A/T-rich S/MARs (aatatt)^35^; A/T rich-2 (atatta/taatat); PBX (atgggc/gcccat); TBX-1 (ttaggg/ccctaa), TBX-2 (aaccct/agggtt, acccta/tagggt), TBX-3 (aaaccc/gggttt, ctaggg/ccctag, SBX (aagccc/gggctt)^17^; TCP/site2^16, 21^ (agccca/tgggct, tgggcc/ggccca); REG (aggccc/gggcct)^17, 18^. For more detailed descriptions see Supplementary Table 8.

Therefore, we tested all possible hexamers for enrichment in 5′ upstream regions of the genes whose expression levels changed in a fold-change-specific manner in the RNA-Seq data. We adapted the two-step procedure for estimation of statistical significance of a fold-change-specific response for the motif enrichment study (Materials and Methods). First, we identified the hexamers significantly enriched in 5′ upstream regions of auxin-responsive genes using the whole-genome data, which yielded 41 hexamers - 31 in DGs and 11 in UGs (Supplementary Table 8). Second, we tested fold-change-specificity of the auxin response for genes with these hexamers in their upstream regions, which returned nine hexamers significantly enriched in the gene groups upregulated by auxin within the same interval of fold changes (Fig. 7). The remaining motifs were non-fold-change-specifically enriched.

Among the hexamers identified, many were known, but none were previously associated with auxin (Fig. 7; Supplementary Table 8). We identified TBX-3 (aaaccc, ccctag), TBX-2 (aaccct, acccta), SBX (gggctt)^15^, TCP/site2 (tgggct, tgggcc)^16^ and REG (aggccc)^17, 18^ specifically enriched in very weakly upregulated genes. TBX-2 (aaccct, acccta)^15^ was associated with a bit wider fold change interval (very weak to weak). An interesting A/T-rich motif taatat, was significantly enriched for genes very strongly upregulated by auxin, but it also enriched not fold-change-specifically for DGs. The motif has been described previously as a cis-element in promoters of plant histone genes specifically bound by proteins activating histone expression during the G1/S transition^19^.

We can conclude that the presence of specific cis-elements in gene promoters may mediate fold-change-specific transcriptional shifts in response to auxin. The question for further study arises: which transcription factors mediate the fold-change-specific response via found cis-elements?

## Discussion

Coordinated expression of genes belonging to the same pathways has been repeatedly suggested^2, 3, 6, 7^. Here, we provide evidence that for some pathways, the involved genes respond to a factor not only unidirectionally but also within a certain interval of fold changes. The factor we study here is auxin, a phytohormone that simultaneously regulates many molecular-genetic processes in a cell, but there is no understanding of how it diversifies its effects on different processes.

We utilized GO^20^ and developed a bioinformatics method to estimate fold-change-specific transcriptional responses (see Materials and Methods). Intriguingly, we found many GO terms specifically overrepresented in genes with similar folds of response to auxin (Supplementary Table 4; Fig. 4, Supplementary Figs 1 and 2). These findings suggest a fine regulation of genes participating in the same biological processes (or having the same molecular functions or associated with the same cell components).

We obtained some initial insight that fold-change-specific response might be mediated by special transcription factors, whose footprints we detected as the hexamers enriched in the upstream regions of genes fold-change-specifically upregulated by auxin (Fig. 7). Presumably, these transcription factors mediate the secondary auxin response, which is still poorly understood. Among the hexamers associated with transcription activation in response to auxin, there is a so-called PBX/TBX/SBX module, a composition of three cis-elements that controls condition-specific diurnal and circadian transcription^15^. In our data, PBX and TBX-1 did not show fold specificity; TBX-2, TBX-3 and SBX were associated with weak auxin response. These results agree with previously reported data on the molecular mechanisms synchronizing cell cycle with protein synthesis^15^, but we also found that auxin plays a major role in regulation of both processes and their connectivity.

In addition to the motifs, whose fold-change-specific response associations might be explained by transcriptome cycling, we identified hexamers that might mediate fold-change-specific transcription irrespective of diurnal rhythms. Namely, very weakly regulated UGs were also enriched for TCP/site2 and REG motifs (Fig. 7). TCP/site2, the binding site for AtTCP20^16^, was enriched in upstream regions of the genes encoding cytoplasmic, mitochondrial and chloroplast ribosomal proteins^21^. The transcription factor for REG was unknown, but this cis-element peaked in the proximal promoter^17^ and was overrepresented in salicylic acid-repressed genes^18^.

Although various aspects of auxin’s influence on cell metabolism in plant roots have been described earlier^3, 6, 7^, applying the fold-change-specific approach for functional annotation made the auxin regulation map more comprehensive. This approach revealed that auxin realizes a well-balanced and thrifty strategy that allows a cell to save its resources during division. On the system level, an increase in auxin levels leads to mobilization of two processes in the root cell: gene expression and cell proliferation, whereas almost all processes not corresponding to these purposes are inhibited (Fig. 4). We showed that gene expression regulation has three bottlenecks tightly regulated by auxin (Fig. 5). The first is at the level of epigenetic regulation of transcription, where auxin fold-change-specifically (from weak to strong levels) upregulates genes related to histone and chromatin modification. Histone modification is a key step in the auxin signaling pathway, as Aux/IAA proteins recruit TPL/TPR histone deacetylases to repress ARF-mediated transcription^2, 22^ Long-term treatment with a histone deacetylase inhibitor trichostatin A (TSA) completely blocked the auxin response in roots^23^.

The second bottleneck is at the level of translation, which auxin activates only weakly. Recently, mutants in subunits of *Arabidopsis* Elongator, a protein complex modulating translational efficiency via maturation of tRNAs, have been shown to exhibit defects in auxin-controlled developmental processes^24^. Our pipeline suggests that RNA methylation is the third stage of gene expression machinery tightly regulated by auxin. Indeed, RNA methylation plays an important role in gene expression by stabilizing transcripts^25, 26^ however, the result might be influenced by mis-annotation of proteins mediating DNA methylation.

## Conclusion

Here, we developed a novel bioinformatics approach to identify coordination in the response trajectories of biological processes. The application of this approach to an auxin-responsive transcriptome provides a more comprehensive map of auxin action in the root cell and shows the presence of bottlenecks in auxin-regulated gene expression. Coordination in fold changes of auxin response in gene transcription can be explained by involvement of yet-uncharacterized transcription factors whose binding sites we detected as overrepresented fold-change-specific cis-regulatory motifs. The next questions involve how such a system unwraps over time and what would occur if the fold-change-specific response was partly disrupted.

## Materials and Methods

### Plant growth and treatment conditions

*Arabidopsis* Col-0 seeds were surface-sterilized and vernalized for 7 days at 4 °C. Seedlings were grown vertically at 22 °C and 150 µmol m^−2^ s^−1^ under a 16-h light/8-h dark cycle on 1/2 Murashige and Skoog (MS) media (Sigma). Three days after germination (dag), seedlings were incubated in liquid 1/2 MS and 1/2 MS supplemented with 1 µM IAA for 6 h. Roots were collected in liquid nitrogen for three biological replicates. Each replicate contained approximately 100 roots.

### RNA preparation

Total RNA from the roots was extracted using TRIzol^®^ Reagent (Ambion, Cat. Num. 15596-018) and DNaseI (Promega), and total RNA yields were measured using an Invitrogen^®^ Qubit™ fluorometer and Agilent Bioanalyzer 2100 microfluidics (Agilent, Santa Clara, CA). Complementary DNA was synthesized using a Revert Aid First Strand cDNA Synthesis Kit (Thermo SCIENTIFIC, USA).

### Transcriptome sequencing

Ribosomal RNA depletion was performed using a RiboMinus™ Plant Kit for RNA-Seq (Invitrogen™), according to the manufacturer’s instructions. Five micrograms of RNA with an RNA integrity number (RIN) of 7.9–9.0 was used. Three hundred nanograms of ribo-depleted RNA was taken for SOLiD cDNA barcoded fragment library construction using a SOLiD™ Total RNA-Seq Kit (Applied Biosystems™). cDNA libraries were amplified by PCR with 16 cycles following the recommendations of the sample preparation protocol. Library concentrations and quality were assessed using an Agilent Bioanalyzer 2100 (Agilent Technologies, USA) and had an average length of ~250 bp. e-PCR (emulsion quantitative polymerase reaction) was performed with a library concentration of 60 pmol. After enrichment, 40 M beads from individual samples were combined, six per line, and were sequenced using SOLiD5500 technology (Applied Biosystems, USA) with a 60-bp read length. From 16 M to 26 M high-quality reads for individual samples were collected.

### Primer design and qRT-PCR analysis

Primers for qPCR were designed using PrimerQuest and PrimerBLAST (Supplementary Table 2). qRT-PCR was performed using an EvaGreen supermix (Bio-Rad) and a CFX-96 real-time thermal cycler (BIO-RAD, USA).

### RNA-Seq data preprocessing

Raw reads were mapped onto the *Arabidopsis* genome (TAIR10) using SHRiMP (v2.2.3)^27^. Uniquely mapped reads were counted using the htseq-count program with the annotation file from TAIR10^28^. The DEGs were detected using the Bioconductor package DESeq^29^. As we investigated specificity in response to auxin, we filtered out the transcripts whose fold changes tended to zero or infinity. The genes were defined as DEGs if the Benjamini–Hochberg adjusted p-value was smaller than 0.05. Further functional annotation analysis was performed with 789 genes significantly upregulated and 659 genes downregulated by auxin.

### Microarray data processing

Time course microarray data with different durations of auxin treatment (0, 0.5, 1, 2, 4, 8, 12, 24 h)^7^ were taken from the Gene Expression Omnibus (GEO) database. The genes were defined as DEGs if the Benjamini–Hochberg adjusted p-value was smaller than 0.05. Only 4-, 8- and 12-h datasets were taken for further analysis, as they showed more than 100 DEGs.

### Functional enrichment analysis to study fold-change-specific responses

To perform functional annotation of the genes with similar folds of response, DEGs in the RNA-Seq data were ranked by fold-changes and subdivided into three, four, six or eight gene subsets that were equal in size (quantiles). UGs and DGs were analyzed separately.

By uniting the neighboring quantiles, for example, quantiles with very weak and weak fold changes or very weak, weak and moderate changes, gene subsets integrating several fold change intervals were created (Fig. 2A). Separately, UG and DG groups in 6-quantile calculations formed 21 gene sets, including the whole UGs or DGs set. Functional annotation of the resulting gene sets was performed using the AgriGO SEA tool^12^ with Fisher’s exact test. To account for multiple testing, a Bonferroni correction was applied for *p*-values, adjusting them by the number of GO terms and the number of tested intervals. The GO terms that were significantly enriched (adjusted *p*-value < 0.001) for at least one gene set were selected for further analysis.

For each pre-selected GO term and each fold change interval, we analyzed the classical contingency table (Table 1) by Fisher’s exact test. If the minimal *p*-value over all 20-fold intervals was lower than the threshold for the multiple test correction (Fisher’s exact test, Bonferroni corrected for the GO terms and the number of intervals), the GO term was considered as fold-change-specifically regulated by auxin. The remaining terms were considered to be not fold-change-specific.

The analysis for 6-quantiles was described in detail in the Results section, and the exact fold thresholds are shown in Fig. 2A. The robustness of this procedure was studied in the tests with 3-, 4- and 8-quantiles. Despite the numbers of fold-change-specific GO terms differed in these tests (Supplementary Table 5), the meaningful results were similar to 6-quantiles (Figs 3–5; Supplementary Figs 1 and 2).

### Search for cis-regulatory motifs associated with the fold-change-specific response

Putative cis-regulatory elements were analyzed in [−1500; 5′ untranslated region (UTR)] regulatory regions taken from TAIR10. We searched for 2080 independent hexamers within these regions. From a total of 4^6^ = 4096 possible hexamers, complementary pairs were considered to be one variant, e.g., aaccaa/ttggtt, and there were some self-complementary hexamers, e.g., aaattt. Enrichment of a hexamer in the gene set responding to auxin within a certain interval of fold changes was estimated by a similar two-step procedure: (1) versus the whole genome set of 5′ upstream regions and (2) versus the 5′ upstream regions of all UGs or DGs. To account for multiple testing, Bonferroni corrections were applied at each step for the number of independent hexamers and the number of tested intervals.

### Data availability

Preprocessed RNA-Seq data for auxin treatment (6 h, 1 µM IAA) in *Arabidopsis thaliana* roots is in Supplementary Table 1. The raw data is available at NCBI Gene Expression Omnibus, accession GSE97258.

## Electronic supplementary material

Supplementary Table 1

Supplementary Table 4

Supplementary Table 5

Supplementary Table 6

Supplementary Table 7

Supplementary Table 8

Additional information
