# Supplementary material for: Auxin regulates functional gene groups in a fold-change-specific manner in Arabidopsis thaliana roots
Source: Sci Rep. 2017 May 30;7:2489. doi: 10.1038/s41598-017-02476-8 (PMC5449405; doi:10.1038/s41598-017-02476-8)
Supplement: Supplementary file 7 — Additional information [file 41598_2017_2476_MOESM7_ESM.pdf]

Additional Information

Auxin regulates functional gene groups in a fold-change-specific manner in *Arabidopsis thaliana* roots

Omelyanchuk NA[1,2,#], *Wiebe DS*[1,2,#], *Novikova DD*[1,2], *Levitsky VG*[1,2], *Klimova N*[1], *Gorelova V*[1,3], *Weinholdt C*[4], *Vasiliev GV*[1], *Zemlyanskaya EV*[1,2], *Kolchanov NA*[1,2], *Kochetov AV*[1,2], *Grosse I*[2,4,5,*], *Mironova VV*[1,2,*]

[1]Institute of Cytology and Genetics SB RAS, Novosibirsk, Russia
[2]LCT&EB, Novosibirsk State University, Novosibirsk, Russia
[3]Ghent University, Department of Physiology, Laboratory of Functional Plant Biology, Belgium
[4]Institute of Computer Science, Martin-Luther-University Halle-Wittenberg, Germany
[5]German Centre for Integrative Biodiversity Research (iDiv), Halle-Jena-Leipzig, Leipzig, Germany
#equal contribution
*corresponding authors: grosse@informatik.uni-halle.de
victoria.v.mironova@gmail.com

Supplementary Table 2. Primers sequences used in qPCR

Gene ID	Gene name	Forward	Reverse
AT4G37390	YDK	CCATAATTCCGCTCCACAGT	ACGCATTCTCCACTGCTTTT
AT3G25190	Nodulin-like21	GAACATAAGACATCACTATCAGCA	GTAAGAGCCATAGCCATCCA
AT3G15540	IAA19	GAGCATGGATGGTGTGCCTTAT	TTCGCAGTTGTCACCATCTTTC
AT1G32450	NRT1.5	AACGTCGACACGATGAAGAAGAAAGAA	TTGGTCGACTTTAGAATCCTTCTCTCG
AT1G69530	EXPA1	TTCTTGTTTATTGCTACCCTTGGAG	TACTACAACACGTCAATACCACACG
AT1G15580	IAA5	CCGCTCTGCAAATTCTGTTC	TCTCCAGCAAGCATCCAATC
AT1G23080	PIN7	TATTGGATTACGTGGAGACCTATTG	CAAGATTGCGGGATGAACATTATAC
AT5G44020	HAD	CTTTGAAAGCTCCCAACCTAAAC	TGGACGAACCATACACATTCC
AT1G17060	SOB7	AAAACACGAACTCTTTCCAA	TCTCCTCCTCTTTTGTCTCA
AT2G39700	ATHER	GCAATTCTATTTACCACATTTGTTC	TTCTCGAAACTCGCCTTAAAC
AT3G61430	ATPIP1	TCTCCTTTGGAACTTCTTCCTTG	CTGGCCTTGTCCTTAGTTGCTTC
AT5G56870	BGAL	TGAGTGTTGCAGTGGGTCTC	GTCCATCTCACACCGGAACT
AT1G75750	GASA1	CTCTTATCGCTTCTCTTCTCATATCTCTT	ACCATTTTTCTTCTGTGAGTTTTCG
AT5G57090	PIN2	CTGGTCTTGGAATGGCTATG	CATCGCAAACCCTGCTACTG
AT5G25350	EBF2	CTCAGTCAATGACCTTGACATCTC	GTAGAGTATATCGCACCTCCATAG
AT4G34110	PAR2	GGGTCGGATGTTCCGGTATC	GGAGCACTTCAGTCTGGTCC
AT3G04720	AtPR4, HEL	TCTGACCATTGCGGTGGATAAGGC	CTCAGTCAATGACCTTGACATCTC
AT4G35100	PIP3	GCTCCTCTTCTCGACATGGGTGAG	CTTGTGGCCGATGACAGTAGCGAC
AT4G14560	IAA1	CGGTTAGATCTCACTGGAGGCCAT	ATCTGCTCCTCCTCCTGCAAAAAC
AT5G20820	SAUR75	CCGCTCTTCCAGCAGCTA	AACTTCGCACGACACAGAGAT
AT4G01630	Expansin	CAGTTTCTGCTGGTTGGTTG	ATAACCACAAGCTCCACCCATT
AT1G12110	AtNRT1.1	AACAAACACCCCCAAAAACTGCA	CCTTGGAAGTCCCAAGCATC
AT1G02500	SAM1	CTCCCATCGAAGCAGAATGGA	GACCGTGAACTCCTTGAGCA
AT1G77690	LAX3	TCACCATTGCTTCACTCCTTC	AAGCACCATTGTGGTTGGAC
AT1G70940	PIN3	GCGTCAATAAAAACCCGAAAG	GAAGCTCCTTGGCGTCATG
AT1G72360	ERF73/HRE1	TCATGCTTCAGGCTCAGCATCAG	ACGTATCTCAGCCGCCCATCTT
AT2G33790	AGP30	AAGGGCTCCGTTCTAAAGCC	CCATCTCATTTGGGGCAGGT
AT2G39370	MAKR4	CGAGAGTTGCAGACGAAGATT	CAACGGACGAAACCCTAAACTA
AT5G52900	MAKR6	CCATTGTGTAAGAGATTAAGGAGATGTAGA	GTCACAAGACCTTCTCCAATAATAATCA
AT1G32450	NRT1.5	AACGTCGACACGATGAAGAAGAAAGAA	TTGGTCGACTTTAGAATCCTTCTCTCG
AT3G04720	AtPR4,HEL	GACCTCGTGGTCAAGCTTCT	CTACATCCAAATCCAAGCCTC
AT4G35100	PIP3	GCTCCTCTTCTCGACATGGGTGAG	CTTGTGGCCGATGACAGTAGCGAC
AT1G12110	AtNRT1_1	AACAAACACCCCCAAAAACTGCA	CCTTGGAAGTCCCAAGCATC
AT4G01630	Expansin	CAGTTTCTGCTGGTTGGTTG	ATAACCACAAGCTCCACCCATT
AT1G25560	AP2/ERF	CACGCGGAGAAGCATTT	CCACACTTTCCCTGTTCTATC
AT5G13930	Cs	GACTACTACTTCCGCATCAC	CAGAGAAGGAGCCATGTAAG
AT5G61600	ERF104	CAAGCGGCGTTTCAATTAC	CAAGCGGCGTTTCAATTAC
AT3G46600	SLP30	CATCCTCCGGCTACAATATAC	CTTGTTAGGAAGAAGGGTGAA
AT3G19930	STP4	TCCTACTTGGTTTCGGAGT	GCAGGGACACAAGCTAATC

Supplementary Table 3. qPCR analysis data.

Gene ID	Gene name	Expression level*, control	Standart deviation	Expression level*, IAA	Standart deviation	T-test	Log2 fold change
AT4G37390	YDK	-1.72274396	0.217902	3.140770371	0.562023599	0.006875	4.92851321
AT3G15540	IAA19	-1.7351189	0.300426	3.38615628	0.785616244	0.010846	5.24928511
AT1G69530	EXPA1	-3.37736281	0.268162	1.791446849	0.269409906	0.000103	3.33020244
AT1G15580	IAA5	-3.62959743	0.873545	2.263181812	0.289258728	0.00644	5.74340986
AT1G23080	PIN7	-0.54190015	0.580679	2.271323443	0.336178387	0.033348	2.76886894
AT1G17060	SOB7	-0.63321966	0.187217	2.911808762	0.090817007	0.000745	3.5386284
AT2G39700	ATHER	-4.05627399	0.621069	0.422515689	0.167458006	0.008596	4.40358209
AT3G61430	ATPIP1	0.304362854	0.269301	-0.78251649	0.161598466	0.003865	-1.09719947
AT5G56870	BGAL	0.043929624	0.591238	-1.90985217	1.085868728	0.036151	-1.77874834
AT1G75750	GASA1	0.29915429	0.293104	-2.03660066	0.10357497	0.007206	-2.35298576
AT5G57090	PIN2	-0.9357058	0.391074	0.081859263	0.122886769	0.036163	0.98430222
AT5G25350	EBF2	-2.77281059	0.732939	0.948514628	0.130138749	0.008655	3.59834746
AT4G34110	PAR2	-0.43235303	0.376556	0.465041887	0.338376398	0.005512	0.89059051
AT4G14560	IAA1	-0.85993405	0.113792	-0.06759202	0.194211667	0.003419	0.79791963
AT5G20820	SAUR75	-5.26945635	0.996482	0.243039802	0.827284809	0.002986	5.43206149
AT1G77690	LAX3	-2.15160746	0.686551	0.696954756	0.609839024	0.000256	2.82419591
AT1G70940	PIN3	-1.10241001	0.23585	1.015902836	0.322859793	0.000715	2.12957533
AT1G72360	ERF73/HRE1	-0.41342909	0.368376	-0.41252303	0.169501387	0.997384	-0.02483679
AT2G39370	MAKR4	-2.70960487	0.111471	1.569814569	0.243294461	0.000319	4.29057603
AT5G52900	MAKR6	-4.40542229	0.589261	0.840660482	0.739505216	0.00304	5.27517581
AT1G32450	NRT1.5	0.937166972	0.184517	-0.85352573	1.087592843	0.07963	-1.55245615
AT3G04720	AtPR4,HEL	0.599036546	0.309925	-1.58958880	0.317720208	0.005487	-2.18784234
AT4G35100	PIP3	0.862066214	0.147165	-0.35172548	0.282193398	0.004789	-1.20106234
AT1G12110	AtNRT1.1	0.715056996	0.271019	-1.32075384	0.295663252	0.008391	-2.03374971
AT4G01630	Expansin	-1.19410756	0.294521	0.065225107	0.279193983	0.00047	1.25757348
AT1G25560	AP2/ERF	0.126299563	0.110815	0.367896862	0.449710892	0.488406	0.28242268
AT5G13930	Cs	0.051343023	0.111352	-0.91493037	0.485531645	0.050641	-0.91387767
AT5G61600	ERF104	0.106270206	0.406333	0.084539126	0.049112137	0.934241	-0.06035642
AT3G46600	SLP30	0.143571777	0.156479	0.540706899	0.330726498	0.279396	0.41571485
AT3G19930	STP4	-0.00792563	0.330086	-0.27154176	1.043629417	0.770901	-0.0566253

*- data was log2 normalized

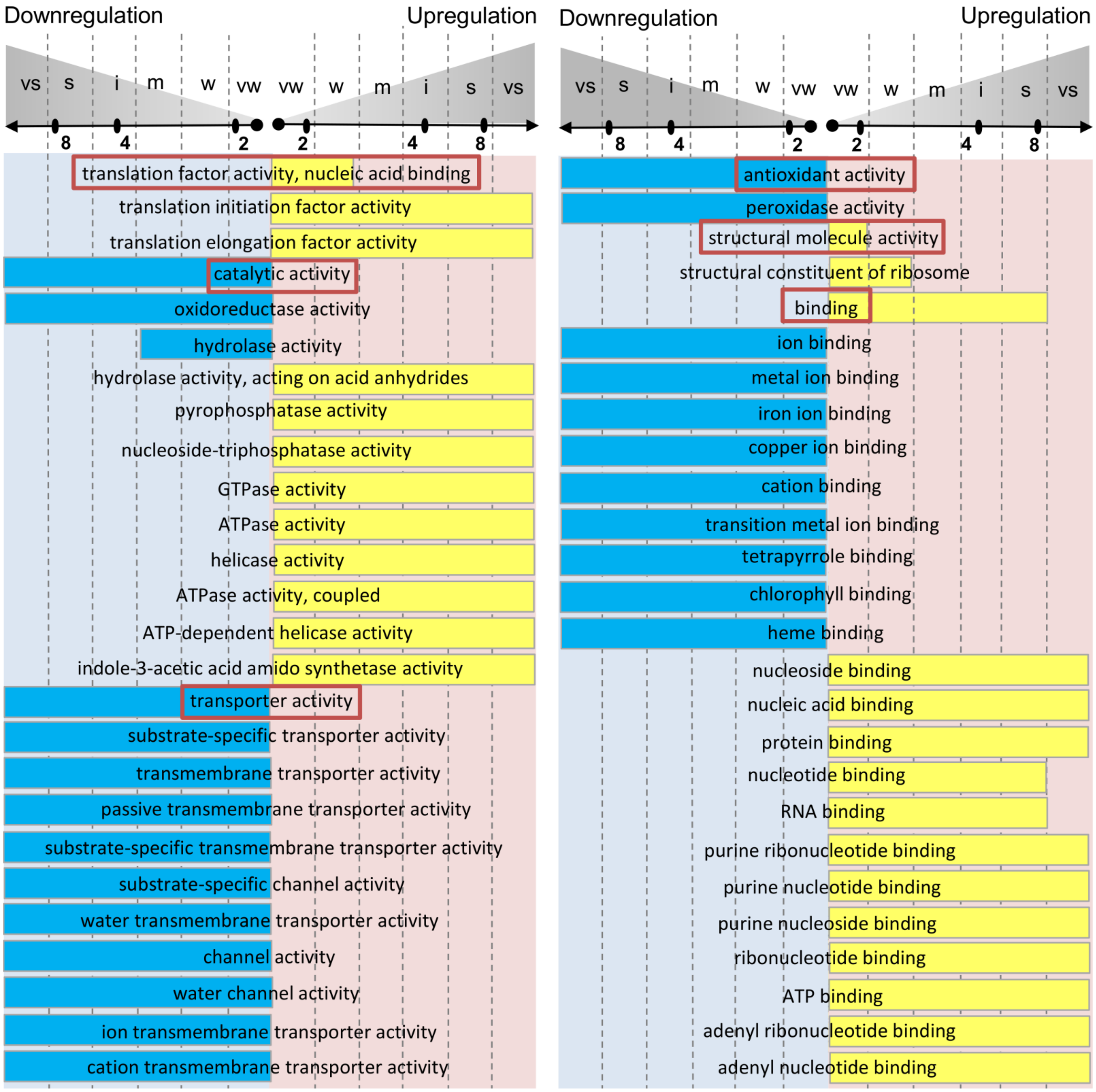

Supplementary figure 1. Auxin regulation of the gene groups encoding proteins with a particular molecular function. For a particular GO term it shows the fold change interval in which significant part of belonging genes responded to auxin. The longest intervals (from very weak to very strong) are not fold-change-specific, while shorter ones are fold-change-specific. Activation of transcription shown by yellow boxes, inhibition of transcription by blue boxes. The representative GO terms were selected from the complete list of significantly enriched in auxin response GO Molecular Function (Supplementary Table 4). Red rectangular highlights the common term for the GO terms group. vs – very strong, s – strong, i – intermediate, m – moderate, w – weak, vw – very weak.

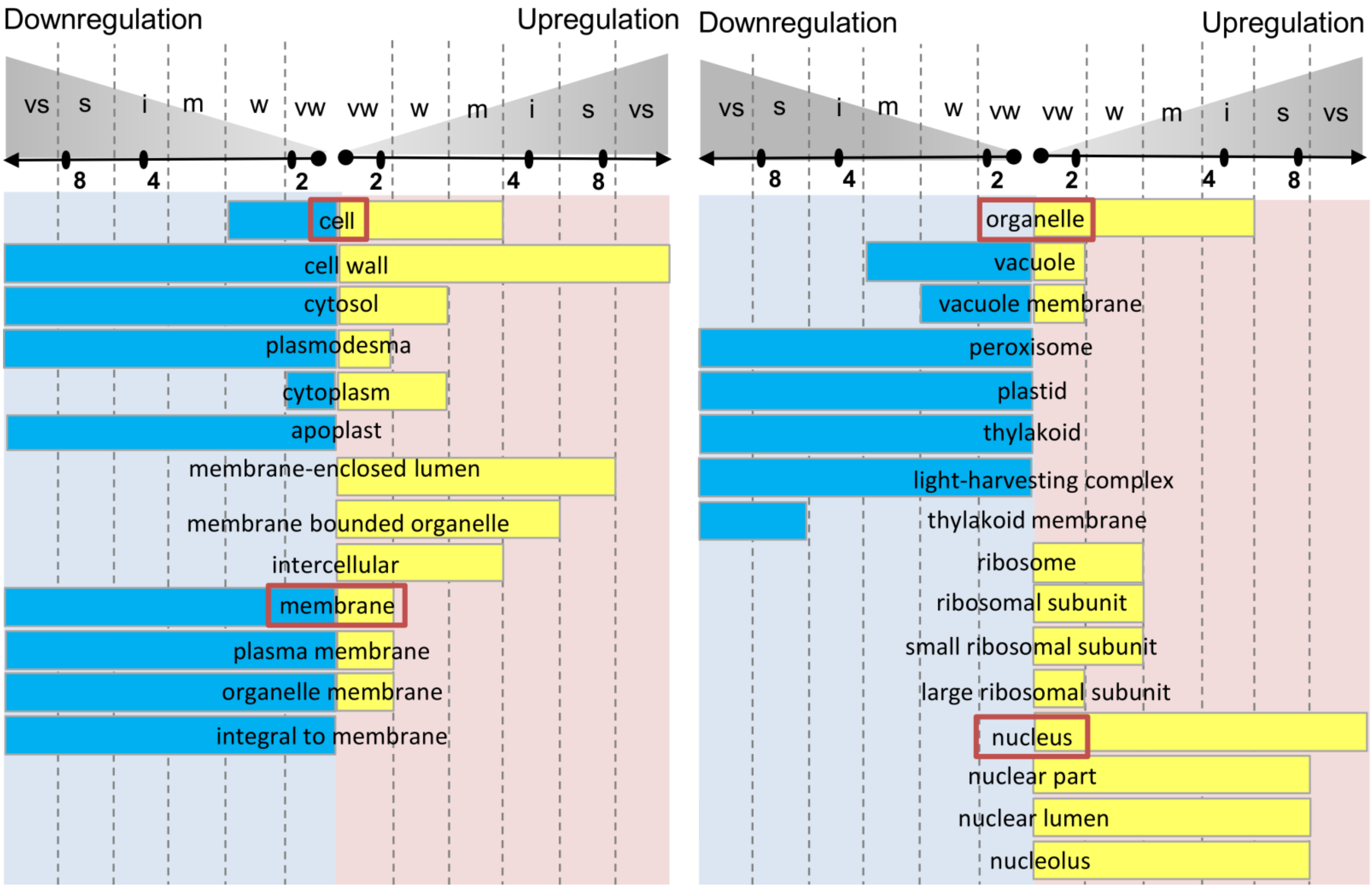

Supplementary figure 2. Auxin differentially regulates genes related to main cellular components. Auxin regulation of the gene groups encoding proteins with a particular molecular function. For a particular GO term it shows the fold change interval in which significant part of belonging genes responded to auxin. The longest intervals (from very weak to very strong) are not fold-change-specific, while shorter ones are fold-change-specific. Activation of transcription shown by yellow boxes, inhibition of transcription by blue boxes. The representative GO terms were selected from the complete list of significantly enriched in auxin response GO Cellular Component (Supplementary Table 4). Red rectangular highlights the common term for the GO terms group. vs – very strong, s – strong, i – intermediate, m – moderate, w – weak, vw – very weak.
